# Precursor RNA structural patterns at SF3B1 mutation sensitive cryptic 3’ splice sites

**DOI:** 10.1080/15476286.2025.2570043

**Published:** 2025-10-08

**Authors:** Austin Herbert, Abigail Hatfield, Alexandra Randazza, Valeria Miyamoto, Katie Palmer, Lela Lackey

**Affiliations:** Department of Genetics and Biochemistry, Center for Human Genetics, Clemson University, Clemson, South Carolina, USA

**Keywords:** RNA structure, 3’ splice site, SF3B1, spliceosome, cryptic splice site, myelodysplastic syndrome

## Abstract

SF3B1 is a core component of the spliceosome involved in branch point recognition and 3’ splice site selection. The SF3B1 K700E mutation (lysine to glutamic acid) is common in myelodysplastic syndrome and other blood disorders. SF3B1 K700E mutants utilize novel cryptic 3’ splice sites; however, the properties distinguishing SF3B1-sensitive splice junctions from other alternatively spliced junctions are unknown. We identify a subset of 192 cryptic 3’ splice junctions with significantly altered use in SF3B1 K700E cells, termed SF3B1-sensitive cryptic 3’ splice sites, and 2800 cryptic 3’ splice sites used in SF3B1 wild-type, termed SF3B1-resistant. We find that SF3B1-sensitive cryptic 3’ splice sites are embedded in extended polypyrimidine tracts. Furthermore, canonical splice sites paired to SF3B1-sensitive cryptic 3’ splice sites are significantly weaker than canonical 3’ splice sites paired to SF3B1-resistant cryptic 3’ splice sites. We test whether SF3B1-sensitive splice sites are structurally different from SF3B1-resistant 3’ splice sites using chemical probing. We develop experimental RNA structure data for 83 SF3B1-sensitive junctions and 39 SF3B1-resistant junctions. We find that the pattern of structural accessibility at the NAG splicing motif in cryptic and canonical 3’ splice sites is similar. However, the magnitude of accessibility differences is less in paired SF3B1-sensitive splice sites than in paired SF3B1-mutant splice sites. Additionally, SF3B1-sensitive splice junctions are more flexible than SF3B1-resistant junctions. Our results suggest that SF3B1-sensitive splice junctions have unique structure and sequence properties, containing poorly differentiated, weak splice sites that lead to altered 3’ splice site recognition in the presence of SF3B1 mutation.

## Introduction

Splicing is ubiquitous across the human transcriptome. More than 95% of human genes contain one or more intronic sequences that must be removed to produce a fully functional mature RNA [1]. These intronic sequences can be processed in multiple ways, through alternative splicing to create multiple isoforms from the same precursor RNA [2,3]. Introns contain several signals that demarcate splice junctions. Core splicing signals include the 5’ splice site, branchpoint, polypyrimidine tract and 3’ splice site [4,5]. These precursor RNA signals are recognized during the initial stages of splicing by the spliceosomal U1 and U2 small nuclear ribonucleoproteins (snRNPs) [6]. In addition, RNA binding proteins (RBPs) can recruit additional splicing factors to guide splice site recognition [7,8]. Mutations in core spliceosome components and major splicing regulators can dramatically alter splicing. Mutations in SF3B1 and U2AF1, core components of the U2 snRNP, broadly alter splicing and are associated with the development of haematopoietic disorders and cancers [9,10]. The most common SF3B1 mutation is a change from lysine to glutamic acid at position 700 (K700E) [11,12]. SF3B1 K700E mutation causes a variety of mis-splicing events, but is specifically associated with cryptic 3’ splicing, a class of alternative splicing where a non-canonical 3’ splice site is recognized. These cryptic 3’ splice sites can be upstream or downstream of the primary canonical 3’ splice site and introduce coding sequence, non-coding sequence and/or premature stop codons [13,14].

All RNAs form co-transcriptional structure based on their primary sequence as they are being transcribed by RNA polymerase [15,16]. RNA structure controls access to sequence motifs that regulate interaction with co-factors like RBPs and ribonucleoprotein (RNP) complexes. RNA folding also creates secondary and tertiary structures that can be recognized by RNA interaction partners. Furthermore, RNA folding can also collapse sequentially distant regions into spatial proximity [17–19]. While RNA structure is clearly important in non-coding RNAs like ribosomal RNA, it has been shown to be an important part of pre-mRNA processing [20,21]. For example, in the *MAPT* precursor RNA, a hairpin at the 5’ splice site spans the U1 interaction motif and is required to control the ratio of exon inclusion and exclusion [22]. However, little is known about precursor RNA structures, and because RNA structure is ubiquitous and dynamic, it is difficult to determine which RNA structures are functionally important [23]. Here, we identify a set of cryptic 3’ splice sites with significant splicing changes in SF3B1 K700E mutants (SF3B1-sensitive or MT C3SS) and characterize them alongside transcripts with cryptic splice junctions with significant use in SF3B1 WT background (SF3B1-resistant or WT C3SS) for sequence and structural features that distinguish sensitive and resistant junctions as well as cryptic and canonical splice sites. We find a consistent pattern of structural accessibility at the 3’ splice site for both cryptic and canonical splice junctions. SF3B1-sensitive junctions, particularly the cryptic splice site, are poorly distinguished structurally from the surrounding intron and have weak splice site scores differentiating them from splice junctions unaffected by SF3B1 K700E mutation.

## Results

### SF3B1 K700E mutation consistently causes mis-splicing at a subset of splice junctions

**Figure 1.**
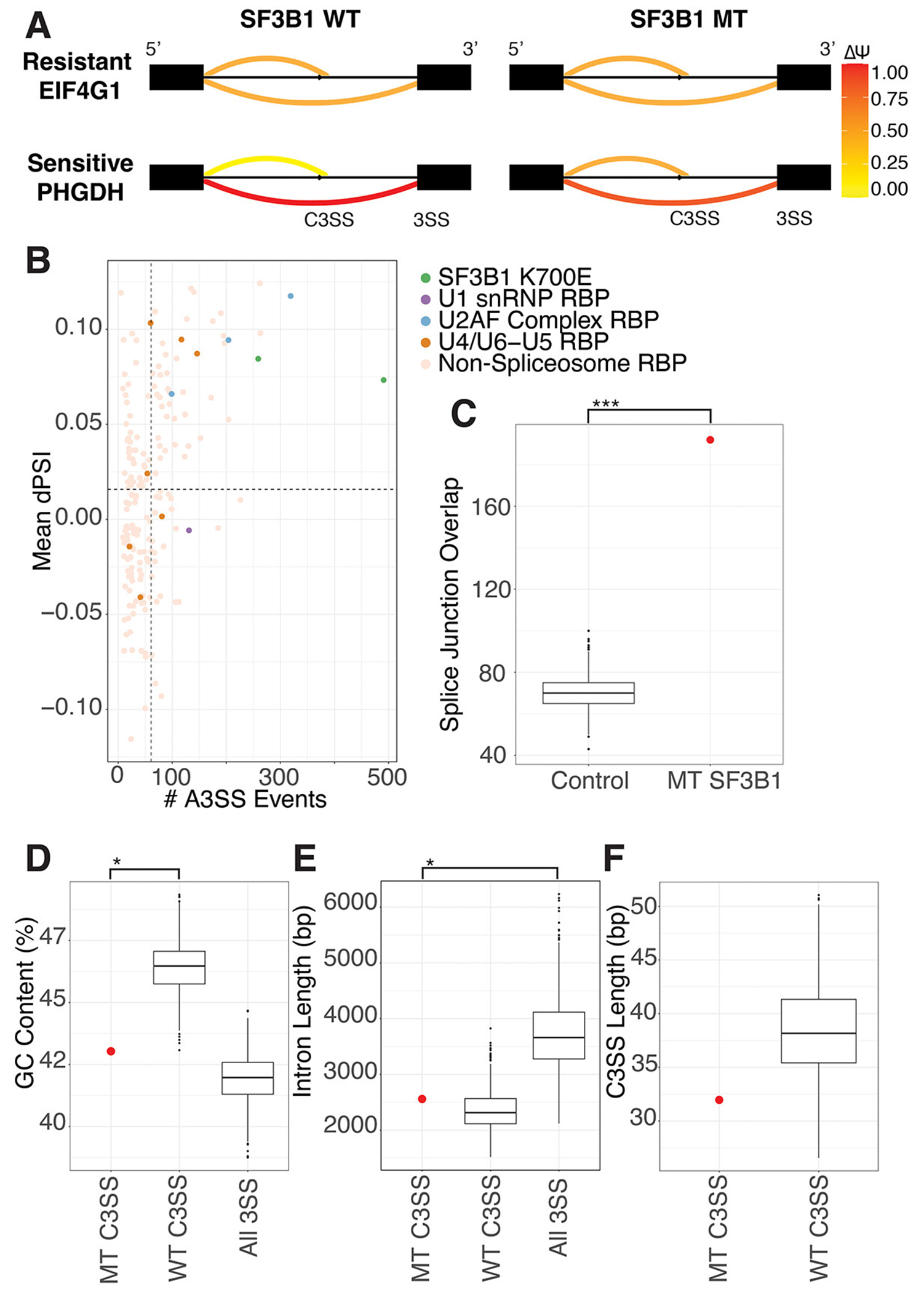
SF3B1 K700E mutation causes consistent mis-splicing at a subset of splice junctions. (A) sashimi plots displaying nomenclature used to denote SF3B1 sensitive (MT) or resistant (WT) cryptic 3’ splice sites (C3SS). SF3B1 resistant C3SS in EIF4G1 displays no change in splicing between SF3B1 normal and K700E cells, however, SF3B1 sensitive C3SS in PHGDH shows significant change in use in SF3B1 K700E cells. (B) degree of alternative 3’ splicing in SF3B1 mutants versus ENCODE RBP knockdown. rMATS analysis from RBP knockdowns in HEPG2 cells were merged and filtered for events with FDR < .09, splice junction counts > 10 in knockdown and controls, and deltaPSI > 0.05 and < −0.05. RBPs known to play a large role in splicing are coloured with different SF3B1 K700E cell types (green), U1 snRNP components (purple), U2AF complex (blue), U4/U6-U5 tri-snRNP complex (orange) and non-splicing RBPs (tan). (C) normalized sets of random splice junctions were tested for their overlap and show significantly fewer alternative 3’ splice site events in common than the overlap between sets of splice junctions from SF3B1 K700E datasets. (D) analysis of GC content in the 150 base pairs upstream of SF3B1 K700E MT-sensitive (MT C3SS) versus WT-resistant cryptic splice sites (WT C3SS). The 192 events shared between MT have significantly lower GC content compared to WT C3SS. WT splice sites selected using random bootstrapping (sampled to 192 events, 1000 times). (E) MT C3SS and WT C3SS have similar intron lengths but are shorter than all other splice junctions. WT C3SS were selected using random bootstrapping. (F) distance in base pairs between C3SS and paired canonical 3SS in SF3B1 MT sensitive versus resistant C3SS analyzed by bootstrapping. The 192 events shared in SF3B1 MT show no significant difference in C3SS length compared to WT C3SS (sampled to 192 events, 1000 times). (*** = p < .0005, ** = p < .005, * = p < .05).

Altering SF3B1 either by knock-down or mutation dramatically changes splicing across the transcriptome (Supp Figure S1A,B). In addition, mis-splicing in SF3B1 K700E mutants is particularly prone to 3’ splice site alteration, resulting in an increase in intron retention and the use of cryptic 3’ splice sites (Figure 1A,B Supp Figure S1C). Although SF3B1 strongly perturbs 3’ splice site recognition in multiple different experiments, increased mis-splicing at the 3’ splice site also occurs after perturbation of other U2 snRNP components, such as components of the U2AF complex (Figure 1B, Supp Figure S1C). Genes that are mis-spliced in heterozygous SF3B1 K700E datasets are not the same as those affected by knockdown or knockout of SF3B1 (Supp Figure S1D). Characteristically, many SF3B1 mis-splicing events are minor changes ( < 0.15) to the percent spliced in (PSI) (Supplemental Figure S1B,C) or are unique to an individual dataset (Supplemental Figure S1D). In addition, mis-spliced genes verified in patients and proposed to have phenotypic effects are not consistently found in all SF3B1 K700E samples (Supplementary Table 1). Small PSI changes and variability between samples has contributed to difficulty in identifying consistently mis-spliced genes across SF3B1 perturbed samples. However, genes that are consistently mis-spliced by SF3B1 mutation may contain a signature responsible for their sensitivity to SF3B1 K700E. (MDS: GSE63569, K562: GSE187356)

To identify a subset of genes that are consistently altered by SF3B1 K700E, we sequenced pre-B NALM-6 cell lines isogenic for SF3B1 WT and SF3B K700E. Our sequencing data shows high similarity to gene expression and splicing patterns from previous sequencing of NALM-6 SF3B1 K700E lines (Supp Figure S1G) [13]. We analysed our NALM-6 sequencing data in combination with RNA-sequencing from published studies in the pre-B K562 cell lines with normal SF3B1 and SF3B1 K700E. For further validation, we compared our cell-line splicing results with data from a set of patients with myelodysplastic syndrome (MDS). Using cell-line and MDS patient datasets allows us to capture natural splicing variation while minimizing the impact of genetic variation in patients and the noise of differential gene expression from different tissues. Due to the disproportionate increase in 3’ cryptic splice site usage with SF3B1 mutation, we concentrate on splice junctions containing a cryptic 3’ splice site for further analysis. We find 192 cryptic 3’ splice sites in 192 genes to be mis-spliced in two or more SF3B1 K700E datasets with a dPSI > .05 using rMATS analysis (Supplementary Table 2). This overlap in number of alternatively spliced genes between three different experiments is significantly more than would be expected by chance (Figure 1C). Both LeafCutter and rMATs splicing analysis produced similar results (Supp Figure S1E). We identified several previously published junctions in our high confidence set, including BCL2L1, MAP3K7, and DVL2 [24–26]. Kegg analysis reveals a significant enrichment of these genes in the nucleocytoplasmic transport pathway (9.9-fold enrichment, Supplementary Table 3) including IPO7, TNPO3 and TPR [27–29]. Interestingly, gene ontology analysis of biological processes reveals a significant enrichment in mRNA transport (6.7-fold) and histone modification (3.2-fold, Supplementary Table 4) [30]. Disruption of SF3B1 results in perturbation of RNA processing, similar to the impact of disrupting many other RNA binding proteins, but for SF3B1, this perturbation may contribute to the diversity of RNA processing in individuals with SF3B1 mutations [31].

### Sequence patterns in SF3B1-sensitive splice junctions

SF3B1 K700E spliceosomes are not known to have strong alternative sequence preferences at the branchpoint or 3’ splice sites (Gupta 2019). To determine whether SF3B1-sensitive MT splice junctions have unique sequence properties, we compared them to control splice junctions containing cryptic 3’ splice sites with similar PSI properties that are SF3B1-resistant (WT junctions). We identified 2801 SF3B1-resistant WT junctions with cryptic splice sites. We found that MT splice junctions and WT splice junctions have significantly different GC content around the 3’ splice site (Figure 1D). Both MT and WT junctions have shorter introns than junctions without alternative 3’ splice sites (Figure 1E). When we measure the distance between the cryptic 3’ splice site to the canonical 3’ splice site, we find that SF3B1-sensitive junctions are not statistically significantly different from WT junctions (Figure 1F). Based on the difference in GC content between MT and WT splice junctions, we decided to further analyse the splice site strength and nucleotide composition at the cryptic and canonical sites of these junctions.

**Figure 2.**
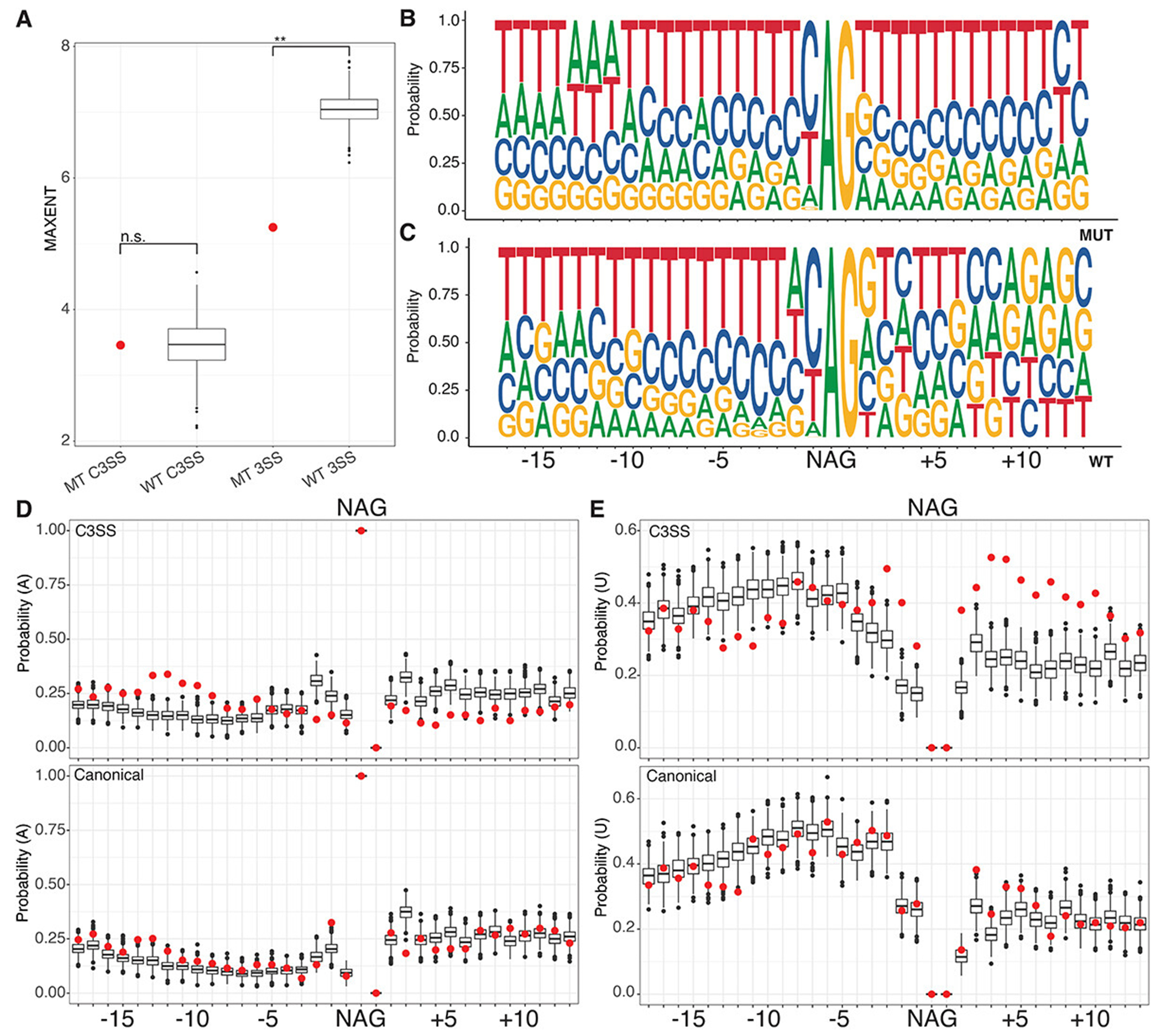
SF3B1 sensitive MT cryptic splice sites are embedded in an extended polypyrimidine tract. (A) splice site strength was calculated using MAXENT at cryptic splice sites (C3SS), with both MT and WT having similar low strengths at the cryptic position. In contrast, the same measurement at canonical splice sites (3SS) is significantly higher at the WT splice site than at MT splice sites. WT splice sites were selected using random bootstrapping. (B) the MT cryptic splice sites (top) are embedded in an extended polypyrimidine tract compared to paired canonical splice sites (bottom) as shown by sequence logo analysis of the nucleotide probability. (D) probability of adenines and (E) uridines flanking MT C3SS (red, top) and paired 3SS (red, bottom) compared to WT C3SS (black, top) and 3SS (black, bottom) indicating changes in nucleotide composition, particularly an increase in uridines downstream from the splice site motif (NAG) at the MT C3SS. WT splice sites were selected using random bootstrapping. (*** = p < .0005, ** = p < .005, * = p < .05).

We analysed the strength of each splice site using the 3’ MAXENT score and found that the WT and MT junctions were significantly different (Figure 2A). This is consistent with our GC content analysis but highlights the fact that while the WT canonical site is much stronger than the MT canonical site, both the WT and MT cryptic splice sites are weak (Figure 2A). The MAXENT score for the 3’ splice site includes the last 20 bases of the intron and first three bases of the exonic sequence [32]. To determine what was driving the differences between the WT and MT scores, we analysed the nucleotide composition around both the cryptic and canonical splice sites. We found that the canonical splice sites of the MT and WT junctions were similar (Supp Figure S2A, Figure 2D,E). In contrast, the MT and WT cryptic splice sites differed in sequence content (Figure 2B,C). The MT cryptic splice site dinucleotide is embedded within an extended polypyrimidine tract that flanks both sides of the junction (Figure 2B), while the WT cryptic site follows a more typical polypyrimidine tract pattern that is U/C rich upstream of the splice site (Figure 2C). Further analysis of nucleotide composition supports sequence differences in the proportion of adenines (A) upstream of the MT and WT cryptic splice sites (Figure 2D, top) but no differences in adenines between MT and WT canonical splice sites (Figure 2D bottom). Likewise, the percentage of uridine (U) is decreased upstream of the cryptic splice site and increased downstream of the cryptic splice site between the MT and WT junctions (Figure 2E, top), but is similar between MT and WT canonical splice sites. These results suggest that the difference between the MT and WT splice scores reflects nucleotide composition outside of the immediate area around the canonical sites (Figure 2D,E, bottom). Although we found a difference in overall GC content, we did not detect a difference in the pattern of G or C nucleotides in the immediate area of the cryptic or canonical splice site dinucleotides (Supp Figure S2A). Our analysis of the sequence features of SF3B1 sensitive junctions supports the hypothesis that genes that are alternatively spliced by SF3B1 mutants have unique sequence properties.

### Conserved accessibility patterns at the 3’ splice site motif

**Figure 3.**
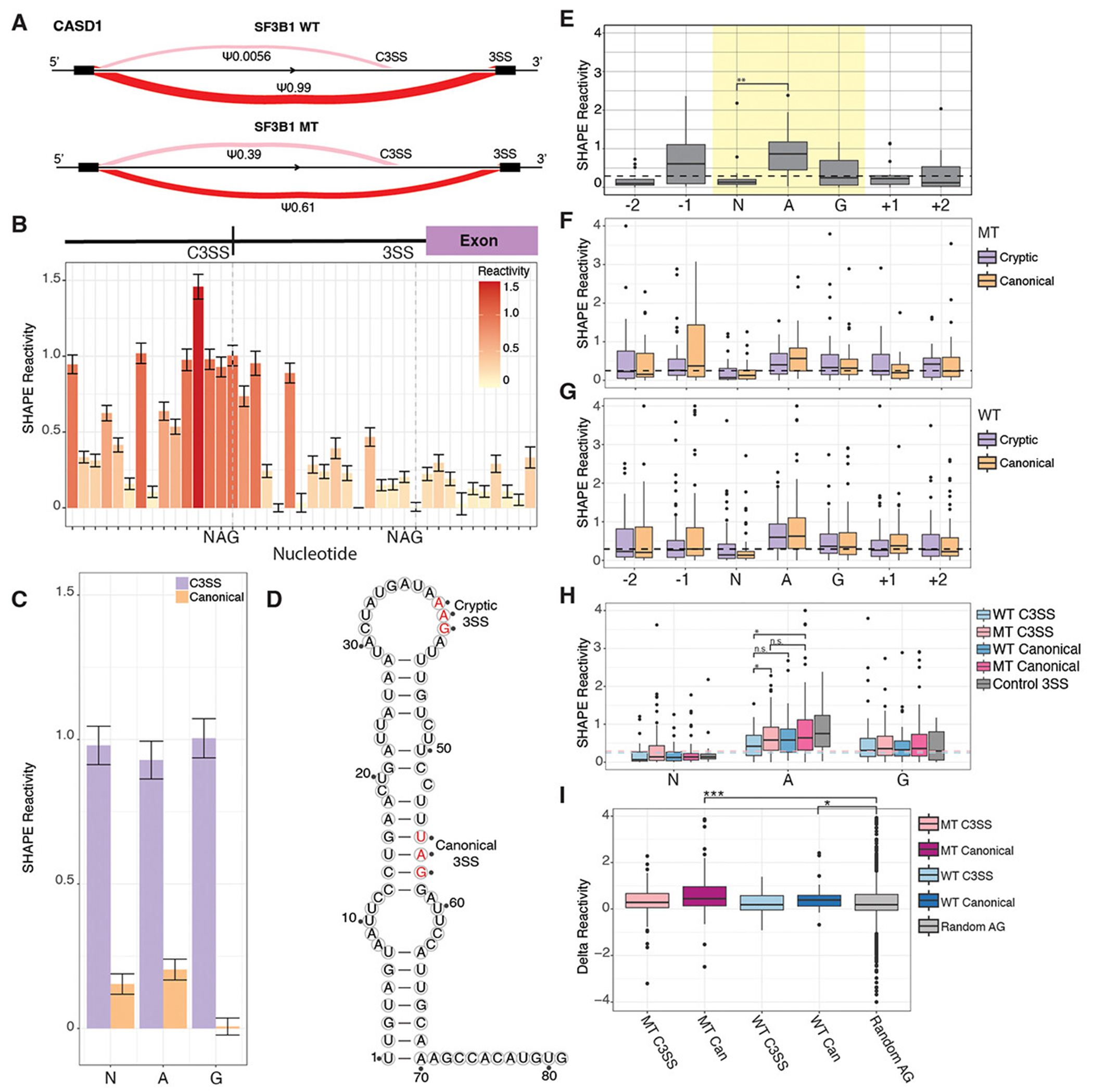
Conserved pattern of accessibility at cryptic and canonical 3’ splice sites. (A) sashimi plot of cryptic 3’ splice site in CASD1 showing a percent spliced in (PSI) of 0.0056 in SF3B1 WT cells and a PSI of 0.39 in SF3B1 K700E mutants. (B) per nucleotide in-vitro SHAPE reactivity and standard error of reactivity at each base show significantly higher reactivity over CASD1 C3SS than paired canonical 3SS. Dashed lines denote C3SS and paired canonical splice sites with NAG labeled on the x-axis. (C) overlayed per nucleotide in-vitro SHAPE reactivity of the CASD1 C3SS (purple) and paired canonical 3SS (orange) illustrates the accessibility difference in SHAPE reactivity between the two splice sites. (D) minimum free energy structure of CASD1 C3SS predicted with in-vitro SHAPE reactivity displays a single stranded C3SS and paired canonical 3SS. Cryptic and canonical 3SS are shown in red text and labeled. (E) control 3’ splice sites (3SS) with no alternative splicing show a characteristic accessibility pattern from chemical probing SHAPE reactivity data with the central adenine in the NAG motif significantly higher than the preceding N. The median reactivity from ±150 surrounding bases is plotted as a reference (black) (*n* = 15). (F) SHAPE reactivity profile of SF3B1 MT sensitive C3SS plotted next to paired canonical SHAPE reactivities and the median reactivity from ±150 splice site bases plotted as a dashed black line (*n* = 83). (G) SHAPE reactivities of WT C3SS and paired canonical splice sites and the median reactivity from ±150 splice site bases plotted as a dashed black line (*n* = 39). (H) SHAPE reactivities from A, B, and C plotted together, significance tested with a pairwise Wilcoxon test, and the median SHAPE reactivities for ±150 splice site bases from MT C3SS (pink) and WT C3SS (blue) plotted as dashed lines. (I) SHAPE reactivities for −120 to + 15 from 3SS normalized to control splice junctions. (*** = p < .0005, ** = p < .005, * = p < .05).

RNA structure influences regulation of pre-RNA processing [23]. We performed chemical probing to determine the *in vitro* structural accessibility for 83 MT splice junctions, 39 WT splice junctions and 15 control splice junctions with no known cryptic 3’ splice sites. We selected these 83 junctions from the core set of 192 SF3B1-sensitive junctions, but not all junctions could be synthesized due to complexity (Figure 3A, Supp Figure S3A). Selected junctions had the same properties as the larger subset used to analyse nucleotide composition (Supp Figure S1F). Base accessibility for individual junctions, like the SF3B1 sensitive junction in *CASD1* (Figure 3A), was assessed via chemical probing of the regions encompassing the cryptic and paired canonical 3’ splice sites (Figure 3B). Overlaying SHAPE reactivity reveals overall differences in accessibility between the NAG motifs of the cryptic and paired canonical 3’ splice site in *CASD1* (Figure 3C). Finally, SHAPE-guided prediction of the minimum free energy structure reveals a single stranded cryptic 3’ splice site and paired canonical 3’ splice trapped in a stem structure (Figure 3D). Replicates of chemical probing experiments resulted in highly correlated reactivity profiles (Supp Figure S3B). To determine whether *in vitro* precursor RNA structure can be used as a proxy for *in vivo* RNA structure, we isolated nuclei and performed chemical probing to develop reactivity profiles across the cryptic and canonical splice sites for five of our splice junctions. All RNAs had high correlation of reactivity data between *in vivo* and *in vitro* conditions (Supp Figure S3B). Thus, we used our *in vitro* data to analyse RNA structure at the cryptic and canonical splice sites in both WT and MT splice junctions. When we quantified the reactivity around the NAG splicing motif, we found a consistent pattern where the N is significantly less reactive than average across the region, the adenine is highly reactive, and the guanine is average (Figure 3E, yellow highlight). The same pattern is visible for the cryptic and canonical splice sites in both MT (Figure 3F, purple and orange) and WT splice junctions (Figure 3G, purple and orange) with the N significantly less reactive than the adenine.

Prior computational predictions have suggested that the guanines within cryptic MT junctions are less accessible than canonical MT junctions [33]. Reactivity data from chemical probing experiments directly measures nucleotide accessibility and can be compared to base-pairing probability predictions [34]. We found no significant difference in reactivity at the guanine in cryptic and canonical splice sites in either the MT or WT splice junctions (Figure 3H). However, the central adenine of the MT cryptic site is significantly less reactive than the central adenine of the WT cryptic site (Figure 3H). The MT cryptic site adenine is also less reactive than the MT canonical site (Figure 3H). The same trend is present for WT cryptic site adenine compared to its more accessible partner canonical site (Figure 3H) although this is not significant. In addition, at the NAG splicing motif, we find that the magnitude of difference between the less reactive N and the highly reactive A is lower for cryptic splice sites than for canonical splice sites, with canonical sites being significantly more reactive than non-splice site control NAGs (Figure 3I). While we find the adenine to be more relevant in our experimental data, the principle of a less accessible cryptic splice site is maintained in our results, similar to the prior study [33].

**Figure 4.**
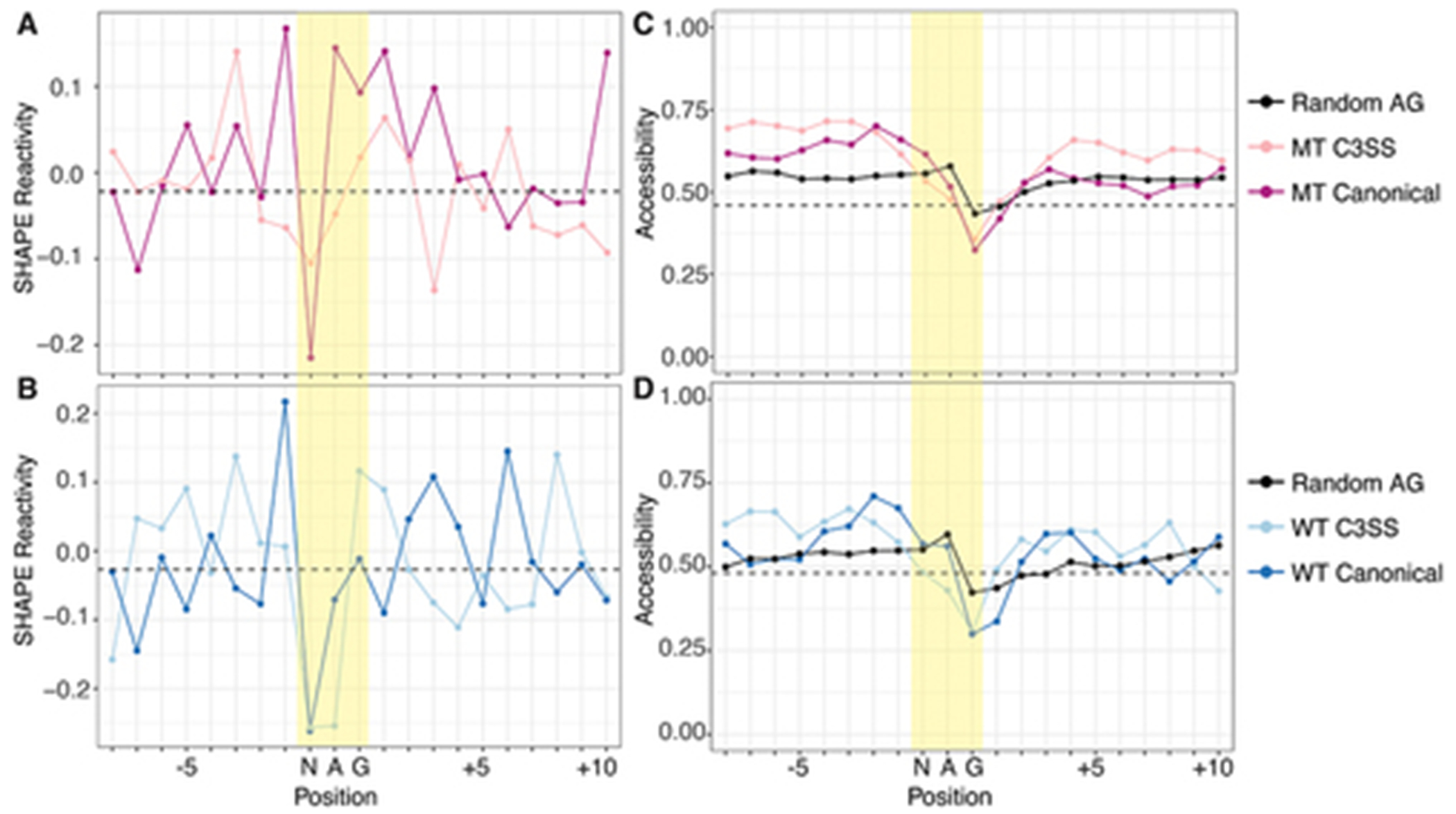
Cryptic and canonical splice sites have similar SHAPE reactivity and predicted accessibility. (A) dip in SHAPE reactivity at the N of the NAG splice site motif for both MT C3SS (light pink), paired canonical (dark pink) and (B) WT C3SS (light blue) and paired canonical (dark blue) compared to flanking nucleotides. Data normalized to mean SHAPE reactivity of random intronic or exon non-splice site AG motifs. Median normalized reactivities for ±10 bases from the MT and WT cryptic splice site plotted as black dashed horizontal lines. (C) base accessibility exhibits a downward trend over the NAG splice site motif compared to flanking nucleotides for both MT C3SS (light pink), paired canonical (dark pink) and (D) WT C3SS (light blue) and paired canonical (dark blue). Base accessibility was determined through SHAPE-guided RNAfold predictions averaged over max distances of 27 to 34.

We normalized our reactivity data to other NAG sequences outside of the annotated splice sites (Figure 4A,B). Non-splice site NAG motifs generally have reactive central adenines, but MT canonical splice sites remain more reactive while the preceding N in the NAG splicing motif is less reactive (Figure 4A). The same pattern is present in the canonical WT splice site although the central adenine is not as reactive (Figure 4B). To further study RNA structure at the 3’ splice site, we incorporated our MT and WT experimental data into computational models of co-transcriptional and post-transcriptional folding. Experimental structural reactivity can significantly improve computational predictions [35]. However, we found that computational co-transcriptional models that fold short sequences (66 nts) can contain artefacts where the 3’ end tends to be more accessible regardless of the sequence (Supp Figure S3C). Folding longer windows of sequence around the 3’ splice site with strict pairing to maintain a model of co-transcriptional folding eliminates this positional bias (Supp Figure S3D). Incorporating reactivity data into a position independent co-transcriptional model, we find cryptic 3’ splice sites trend towards less accessibility than canonical 3’ splice sites (Figure 4C, Supp Figure S3E). Post-transcriptional models follow the same trend with the central adenine of the cryptic splice site less accessible than the canonical splice site in both WT and MT junctions (Supp Figure S3F). Interestingly, other non-splice site NAG sequences within our data share similar high reactivity at the adenine (Figure 4C,D). The pattern of reactivity data, showing inaccessible Ns and reactive As at splice sites, is not reproduced in the base-pairing probabilities after computational modelling, even with experimental data incorporated, suggesting that the sequence context of cryptic and canonical is influencing structure, particularly in the co-transcriptional model with tight distance constraints (Figure 4C,D, Supp Figure S3E). Overall, like in reactivity data, canonical splice sites trend towards higher accessibility than cryptic splice sites (Supp Fig S4A).

**Figure 5.**
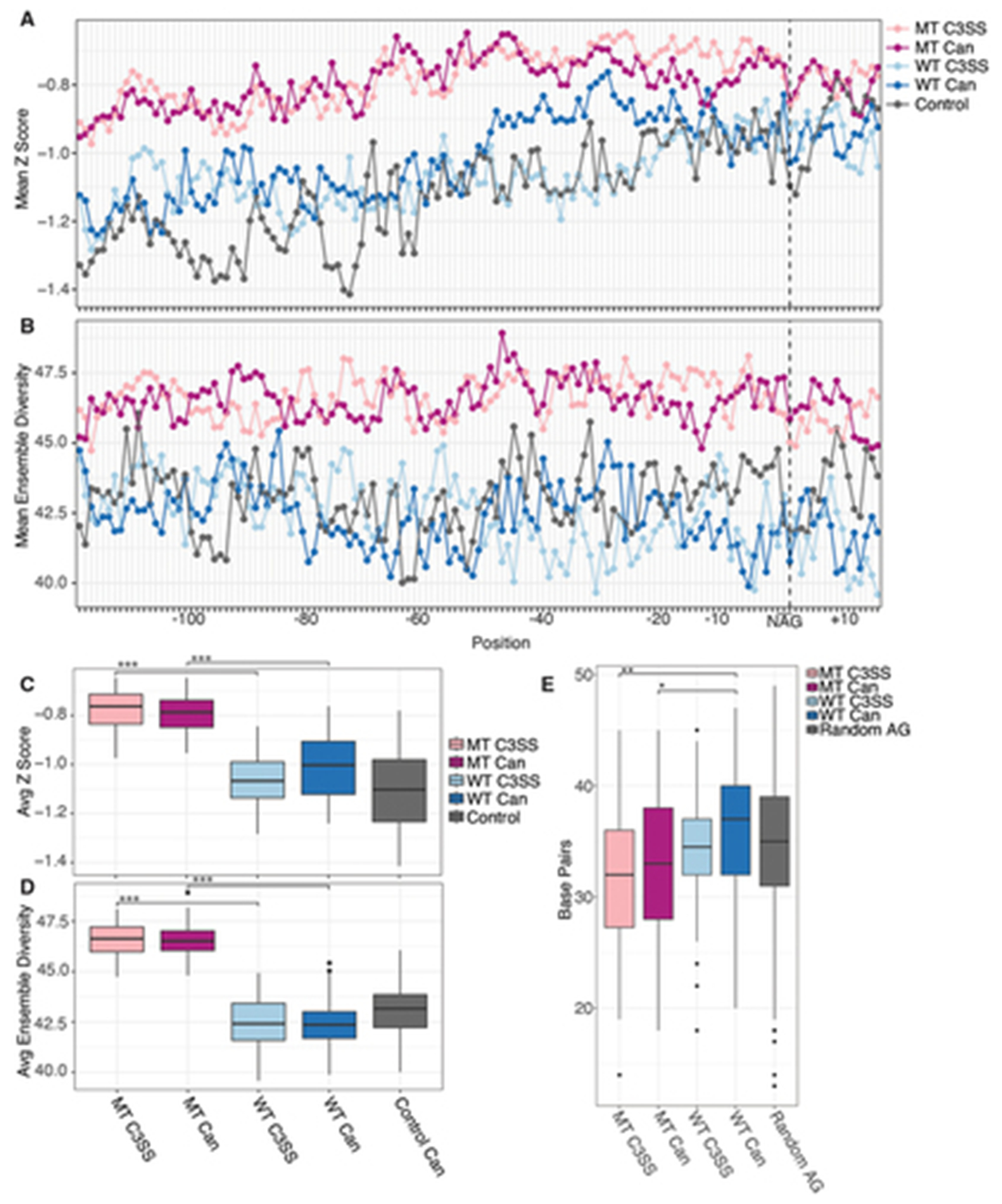
Increased flexibility of upstream intronic sequences in SF3B1 MT sensitive 3’ splice sites. (A) higher Z scores suggest the presence of less stable structures upstream of NAG splice site motifs for MT cryptic 3’ splice sites (MT C3SS, light pink) and paired canonical (MT can, dark pink upstream of NAG splice site motifs compared to WT C3SS (light blue) and WT canonical (dark blue). (B) likewise, higher ensemble diversity indicates the propensity of MT C3SS (light pink) and MT canonical splice sites to form multiple distinct secondary structures compared to WT C3SS (light blue) and paired WT canonical splice sites (dark blue). Control 3SS (gray). (C) significantly less stable MT regions (light pink, dark pink) compared to WT C3SS (light blue) and paired canonical (dark blue). Box plot of Z scores averaged per base for bases −121 to + 15 across all MT C3SS, paired MT canonical, WT C3SS, paired WT canonical and control 3SS (gray). (D) significant difference in ensemble diversity between SF3B1 MT C3SS (light pink) paired MT canonical (dark pink), WT C3SS (light blue) and WT canonical (dark blue). Ensemble diversity plotted as described in (C). (E) significant difference in the number of paired bases in MFE structures of MT C3SS (light pink) and MT canonical (dark pink) compared to WT C3SS (light blue) and WT canonical (dark blue). The number of paired bases for −121 and +15 bases flanking cryptic and canonical splice sites was determined from SHAPE guided MFE structures produced with RNAfold. (*** = p < .0005, ** = p < .005, * = p < .05).

Accessibility at the NAG splice site is limited to only three nucleotides and may not represent the accessibility of the overall junction. To further investigate the surrounding region, we incorporated experimental reactivity data into scanFold to analyse intronic sequences around the splice sites for stable structures and ensemble diversity [36]. We found that MT splice junctions contain significantly less stable structures across both cryptic or canonical splice sites than their partner sites in WT splice junctions (Figure 5A,C). Supporting this observation, MT splice junctions have significantly higher ensemble diversity at both cryptic and canonical splice sites than their partner WT splice sites (Figure 5B,C). Within WT splice junctions that are resistant to splicing changes in the context of SFB31 mutation, cryptic sites are significantly more stable, although this difference is minimal and does not correspond to a significant change in ensemble diversity (Figure 5C,D). To confirm findings made with *in vitro* SHAPE-MAP data, we performed *in vivo* SHAPE-MAP on five SF3B1 MT splice junctions (Supp Figure S5). We generated Scanfold ensemble diversity and Z score calculations using these *in vivo* SHAPE reactivities. *In vitro* and *in vivo* normalized SHAPE reactivities are highly correlated (average Spearman correlation of ρ = .74, Supplementary Table 6). Likewise, ensemble diversity and Z scores show similar trends between structures predicted using *in vitro* and *in vivo* SHAPE reactivities (Supplementary Figure 6).

To corroborate this, we analysed the amount of base-pairing in co-transcriptional minimum free energy structures surrounding the NAG motif. We find that there is significantly less base-pairing around MT junctions than in WT or control junctions at both the cryptic and canonical sites (Figure 5E). This is consistent with our Scanfold results showing increased flexibility around MT junctions (Figure 5C,D). Interestingly, within minimum free energy structures, WT canonical splice sites show more global base-pairing than WT cryptic splice sites (Figure 5E). Overall, this structural data suggests that robust splice junctions have higher global base-pairing, stable local structures and less ensemble diversity (Figure 5C–E).

## Discussion

Here we describe the structural pattern of 137 splice junctions, the majority of which contain a canonical and a cryptic 3’ splice site. This is the largest set of RNA structure data at 3’ splice junctions and is highly correlated to our *in vivo* validation subset. Analysing our reactivity data, we find that 3’ splice junctions have a characteristic pattern of accessibility at the NAG splicing motif where central adenine is more reactive than the preceding N. This pattern is present in both cryptic and canonical sites and also in both MT and WT splice junctions. However, we find that cryptic splice sites have lower magnitude changes in reactivity between the N and the A than non-splice site NAGs. We propose that the NAG pattern of reactivity is necessary to demarcate a strong splice site and that the magnitude of change between the N and the A may distinguish the splice site from the surrounding intron. Computational modelling of introns, even with reactivity data, does not capture the experimental reactivity pattern at the splice site. The transition between low and high reactivity at the N and A nucleotides in the experimental data is abrupt and may be due to lack of flexibility at the nucleotide rather than direct base-pairing and slight differences in nucleotide reactivity [37]. This could account for why the reactivity data is not reflected in the base-pairing probability from computational modelling. However, we find that the pattern of accessibility using computational modelling is similar for cryptic and canonical splice sites in both MT and WT splice junctions, like in our reactivity data. Derivation of experimental reactivity data on intronic sequences is important for direct analysis of nucleotide accessibility and to best inform computational modelling.

In addition to the structural characteristics at the NAG splice site, we also find that MT splice junctions are generally more flexible with higher ensemble diversity, lower structural stability and less base-pairing than WT or control splice junctions. This increase in flexibility covers a large area around the splice junction. While an increase in RNA structure in the WT splice junctions may seem counterintuitive for promoting splicing, there are other examples where RNA structure forms and promotes accessibility of specific regions. For example, *MBNL1* mRNA is bound by its own product, MBLN1 protein, which causes downstream restructuring of a functional RNA element [38]. Likewise, in the 5’ UTR of *SERPINA1* mRNA, structures upstream of the start codon are necessary to promote accessibility of the Kozak sequence [39]. Less flexibility in WT splice junctions may promote splicing or recruit specific RNA binding proteins during early splice site recognition compared to MT splice junctions.

Previous studies have looked for a role of specific mis-spliced genes in oncogenic phenotypes or proposed roles other than splicing in SF3B1 disruption, such as R-loop pathways [40]. We analysed whether MT genes that are consistently mis-spliced by SF3B1 K700E have similar structural characteristics to further understand the mechanism underlying the cohort of mis-spliced genes. We find that MT splice junctions have weak splice site strength at both the cryptic and paired canonical splice sites. This corresponds with a lack of differentiation in reactivity between the splice site NAG nucleotides for MT splice junctions, particularly the cryptic splice site compared to non-splice site NAG motifs. In addition, we find that MT splice junctions are significantly more flexible than WT splice junctions. Our results show that multiple features differentiate MT splice junctions from WT splice junctions, suggesting that recognition of splice junctions is likely due to multiple factors across a broad region of the precursor mRNA encompassing both the cryptic and canonical splice sites.

## Methods

### Cell culture

Human Nalm-6 SF3B1 K700E mutant and wildtype cells are from Horizon Discovery (Lafayette, Colorado). Nalm-6 cells were maintained in RPMI-1640 medium (Gibco) supplemented with 10% foetal bovine serum (FBS), 1% penicillin-streptomycin at 37°C with 5% CO2 and passaged every three days.

### Library preparation and RNA sequencing

Whole cell RNA was extracted from 1x10^6^ SF3B1 K700E and wildtype cells using standard Trizol extraction followed by DNA digestion and rRNA depletion prior to reverse transcription (ThermoFisher TRIzol, 5PRIME PhaseLock Heavy, Invitrogen Purelink RNA columns, ThermoFisher Scientific TurboDNAse Kit, and Qiagen FastSelect -rRNA depletion kit). Briefly, 1 ug of RNA was incubated with QIAseq FastSelect - rRNA HMR in two-minute steps decreasing in 5°C increments from 75°C to 55°C and finishing with 37°C for two minutes followed by 25°C for two minutes. This reaction was put straight into RT. Reverse transcription was performed using SuperScript IV reverse transcription kit with random nonomers, and second strand synthesis was completed with the Second Strand cDNA Synthesis Kit (Invitrogen, ThermoFisher Scientific). cDNA was purified with the Monarch PCR and DNA cleanup kit (New England Biolabs, NEB) and sequencing libraries were prepared under standard protocol using the Nextera DNA Library Preparation Kit (Illumina). RNA purification and cDNA libraries were prepared in triplicate for Nalm-6 SF3B1 mutant and wildtype cells grown on different days. Paired-end libraries (2 ×150) were sequenced on an Illumina NovaSeq.

### Splice junction characterization and quantification

Specific parameters and scripts are available on GitHub (herber4/SF3B1_C3SS_Structure_Paper.git). RNA sequencing reads from MDS patient samples (MDS: GSE63569, SF3B1 K700E *n* = 8, SF3B1 WT *n* = 4, CD34 + healthy *n* = 5) and K562 SF3B1 K700E isogenic cell line (GSE187356, SF3B1 K700E *n* = 2, SF3B1 WT *n* = 2) were downloaded from the Sequence Read Archive [25,41] and our NALM sequencing data all under-went the same analysis pipeline. Bbduk from bbmap was used to trim reads of adapters and to discard low quality reads and any remaining rRNA reads. Quality trimming was performed using bbduk (BBMap v38.73) using parameters provided at (bbduk_trim.sh) [42]. Reads were mapped to Hg38 using Hisat2 v2.1.0 under default parameters and alignment files were sorted and indexed using Samtools v1.10 (hisat_alignment.sh) [43,44]. Splicing analysis was conducted using rMATs v4.1.1 on the SF3B1 K700E samples against their matched controls with parameters allowing for novel splice site detection and variable read length (rmats.sh) [45]. Alternative 3’ splicing events shared in two or more cell types with and FDR < .1 and deltaPSI > .05 were included for further analysis. We identified resistant cryptic 3’ splice sites without splicing changes in SF3B1 mutant cells. First, junctions were extracted from mapped bam files using the regtools junctions extract command allowing for a minimum anchor length of 8 base pairs, and a minimum and maximum intron size of 50 and 500,000, respectively (https://github.com/griffithlab/regtools.git). Then, junctions identified in the previous step were annotated with the Gencode Hg38 v39 gtf file using the regtools junctions annotate command under default parameters. Finally, identification and filtering of cryptic 3’ splice sites in SF3B1 WT cells was conducted in R, resulting in the identification of 2801 C3SS events. These 2801 events were used as controls for all further analyses of sequence characteristics. Events were filtered and compared to previously published ENCODE RBP knockdown rMATS (https://github.com/flemingtonlab/SpliceTools.git) [46]. GC content, intron length, C3SS length, sequence content and bootstrapping analyses were all conducted in R v4.4.2. Maximum entropy scores for cryptic and paired canonical splice sites were generated with MAXENT and further analysed for significance in R [32].

### Gene fragment design, in-vitro & in-vivo SHAPE mapping

Gene fragments were designed and synthesized by Integrated DNA technologies (IDT). Each gene fragment contains 450 base pairs flanking both sides of the 3’ splice site, with a T7 promoter on the 5’ end and both ends capped with stabilizing hairpin sequences. Using the gene fragments as template, in vitro RNA synthesis was performed for two hours at 37°C using the HiScribe T7 High Yield RNA Synthesis Kit (New England Biolabs). In vitro synthesized RNA was DNA digested with TurboDNAse and purified with RNA XP cleanup beads (Beckman Coulter). RNA was incubated in folding buffer (400 mM bicine, 200 mM NaCl, 20 mM MgCL2) and 25 mM 5NIA or DMSO at 37°C for 5 minutes and immediately cleaned up using RNA XP beads (Beckman Coulter) and input into error-prone reverse transcription using the Invitrogen Super Script II (ThermoFisher Scientific) with modified RT buffer (10X: 500 mM Tris pH 8, 750 mM KCl) supplemented with 6 nM manganese, RNAse inhibitor, and reverse primer 5-TGTTGGAGTCACTCGACTCCGGT-3. The RT product was cleaned up with the RNA XP beads and used as template for the Second Strand cDNA Synthesis kit (Invitrogen, ThermoFisher Scientific). Finally, libraries were synthesized with standard Nextera DNA library prep and sequenced (2 ×150, Illumina).

### In vivo validation of RNA structure

Nalm-6 SF3B1 K700E mutant cells were grown to confluency, 1 × 10^7^ cells were pelleted, and nuclei were fractionated using the Cytoplasmic and Nuclear RNA purification kit (Norgen Biotek). Briefly, cell pellets were treated with lysis buffer J on ice for 15 minutes, and nuclei separated by centrifugation at 600 *g* for 5 minutes. Remaining nuclei were resuspended in 1X buffer SK then treated with 1X folding buffer, 200 U murine RNAse inhibitor (NEB) and either 25 mM 5NIA or DMSO and incubated at 37°C for 10 minutes. Finally, RNA was purified with the remaining steps of the Norgen purification kit and DNAse digested as previously described. Error-prone RT was conducted as previously described with the addition of random nonomers instead of gene specific primers, and RT products were cleaned up using the RNA XP beads. To amplify target preRNA, subsequent PCR reactions using gene specific primers were used to incorporate Illumina barcodes (Supplementary Table 5). Both PCR reactions were conducted for 15 cycles each with the Monarch Q5 High-Fidelity DNA Polymerase (NEB). Final PCR products were pooled and size selected from a DNA agarose gel with the Monarch DNA Gel Extraction Kit (NEB). Libraries were sequenced at 2 × 150on an Illumina MiniSeq. SHAPE profiles were generated with shapemapper2 v2.2.0 [47] and technical replicates with high correlation were merged for further analysis. Correlation analysis between in vivo and in vitro SHAPE profiles was conducted in R. Correlation between *in vivo* and *in vitro* SHAPE profiles was further validated by analysing ensemble diversity and Z scores predicted with ScanFold [48]. Scanfold was run with on each gene fragment while providing either *in vivo* or *in vitro* SHAPE reactivities at regions with both data, and NAs filled at remaining nucleotides. Scanfold was run with a window size of 200 nucleotides and step size of 20. Per base z scores and ensemble diversities were compared at each junction and averaged over all splice sites with in vivo data and visualized in R. Significance between nucleotide reactivity was assessed with Wilcoxon test.

### SHAPE analysis

SHAPE analysis was conducted with shapemapper2 v2.2.0 under default parameters using DMSO controls as untreated and 5NIA reads as modified, respectively (shapemapper.sh) [47]. Normalized SHAPE reactivity profiles were adjusted to between 0 and 4 and utilized for all further downstream analyses. SHAPE guided base pairing probabilities for max distances of 27 through 34 were predicted using the RNAfold function of ViennaRNA v2.4.18 and the mean of this window was used for further analysis. Structural trends flanking the cryptic and canonical splice sites were assessed by predicting SHAPE guided MFE structures for the last 120 bases of the intron and the first 15 bases of the exon using RNAFold function with no max distance [49,50]. Dot bracket files produced in the previous step were imported into R, and hairpin length was determined as the sum of paired bases divided by two. Structural trends in this region were further assessed by evaluating Z scores and ensemble diversities predicted with Scanfold [48]. Scanfold was run under default parameters using SHAPE data to guide predictions and allowing a window size of 200 and step size of 20. Windows from both cryptic, canonical and control splice sites were analysed. Per base z scores and ensemble diversities were averaged over all splice sites included in each group and visualized in R. Significance between nucleotide reactivity was assessed with Wilcoxon test.

## Supplementary Material

Supplemental Figure 2

Supplemental Figure 1

Supplemental Figure 3

Supplemental Figure 4

Supplemental Figure 5

Supplemental Figure 6

Supplemental Table 4

Supplemental Table 6

Supplemental Table 5

Supplemental Table 3

Supplemental Table 2

Supplemental Table 1

## Data Availability

Normalized SHAPE reactivities and scripts used for data analysis and visualization are available at https://github.com/herber4/SF3B1_C3SS_Structure_Paper.git. Supplementary tables are available at https://github.com/herber4/SF3B1_C3SS_Structure_Paper/blob/main/Supplementary_Tables_A4_Reduced.xlsx. Raw sequencing reads from NALM-6 SF3B1 K700E and wild-type samples are deposited to the gene expression omnibus: GSE289451. Other data are available upon request.
