# Supplementary figures and images for: Precursor RNA structural patterns at SF3B1 mutation sensitive cryptic 3’ splice sites
Source: RNA Biol. Author manuscript; Available in PMC 2025 Dec 1. (PMC12520072; doi:10.1080/15476286.2025.2570043)

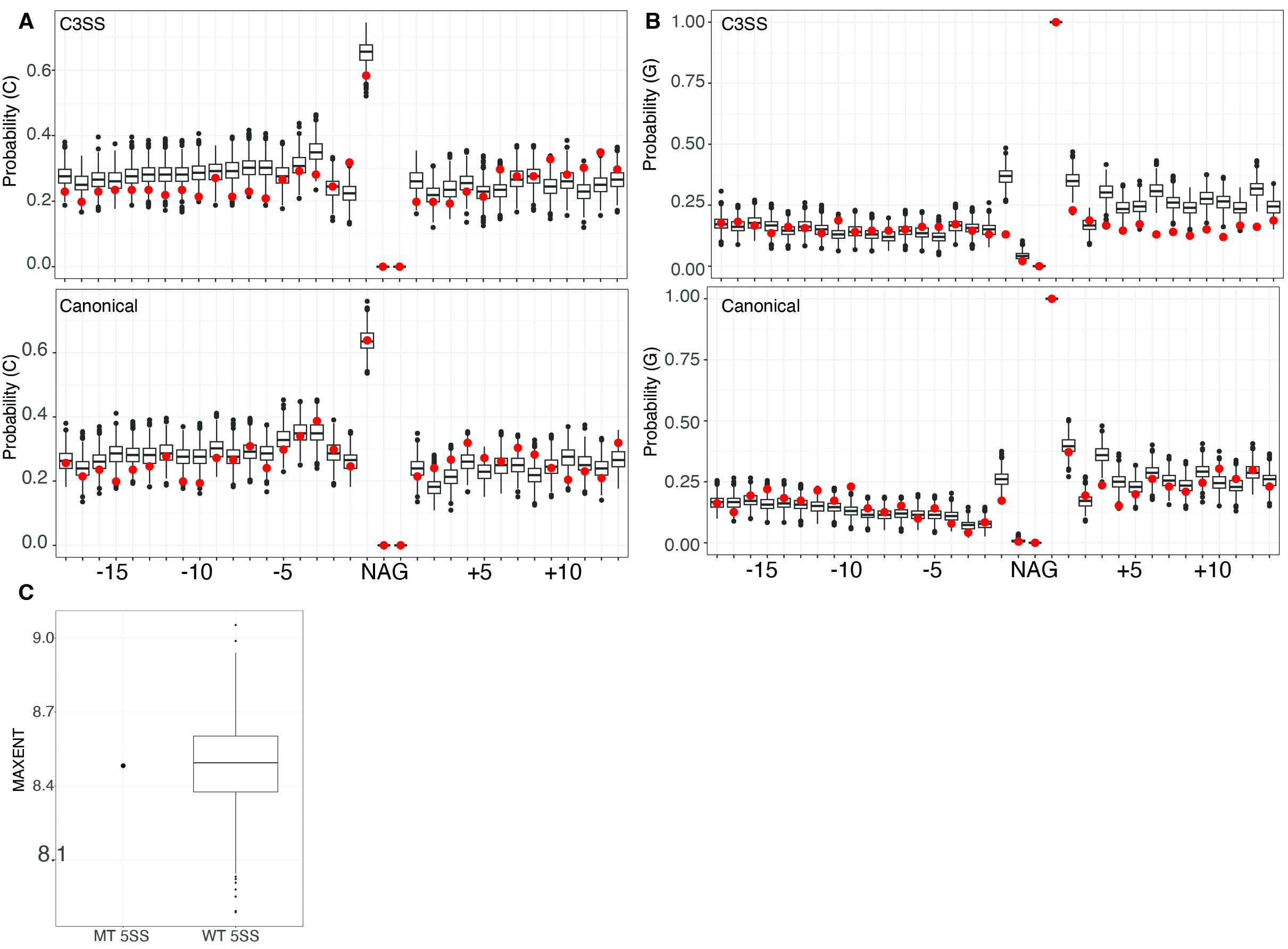

Supplement: Supplemental Figure 2 [file NIHMS2117327-supplement-Supplemental_Figure_2.jpg]

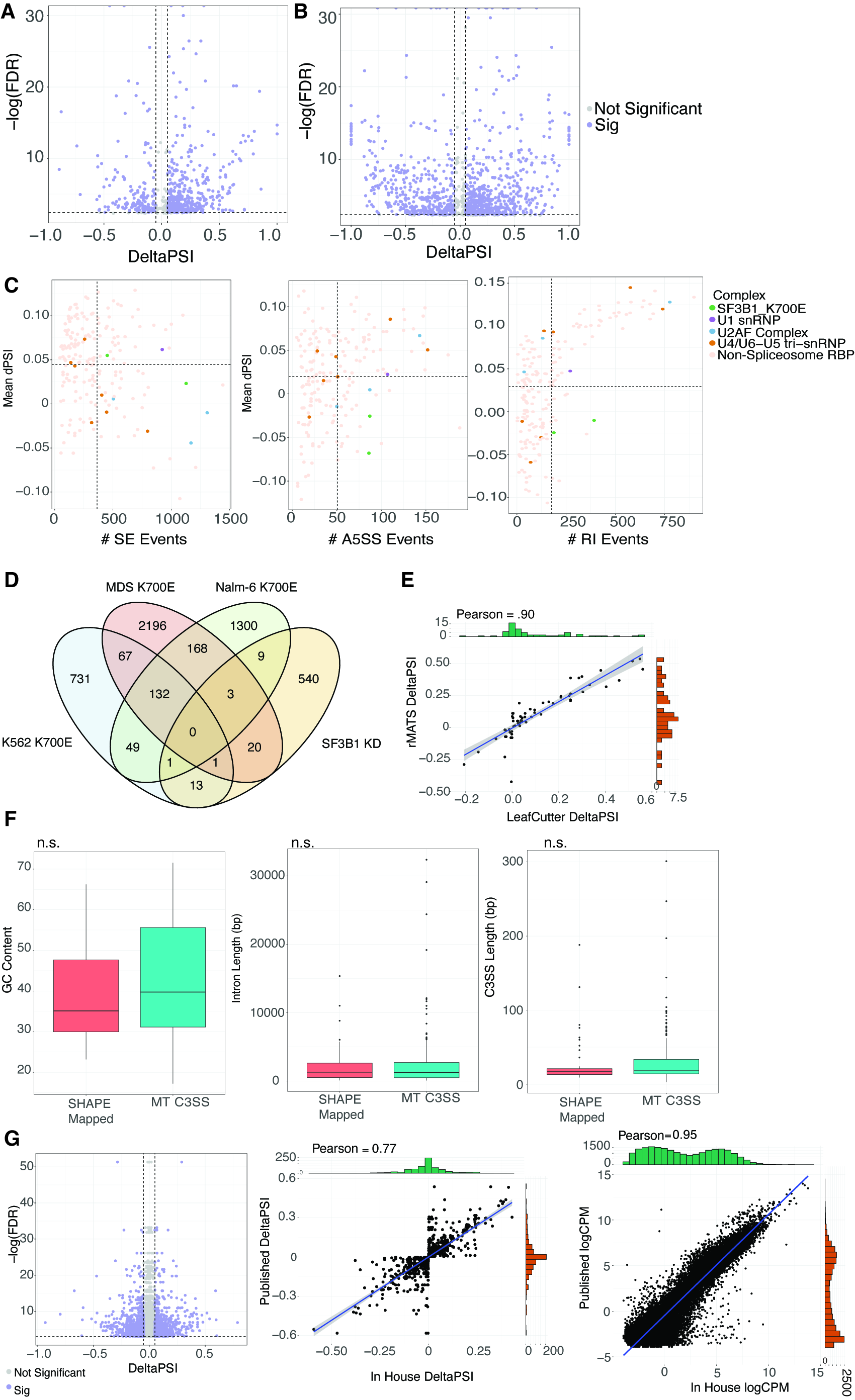

Supplement: Supplemental Figure 1 [file NIHMS2117327-supplement-Supplemental_Figure_1.tif]

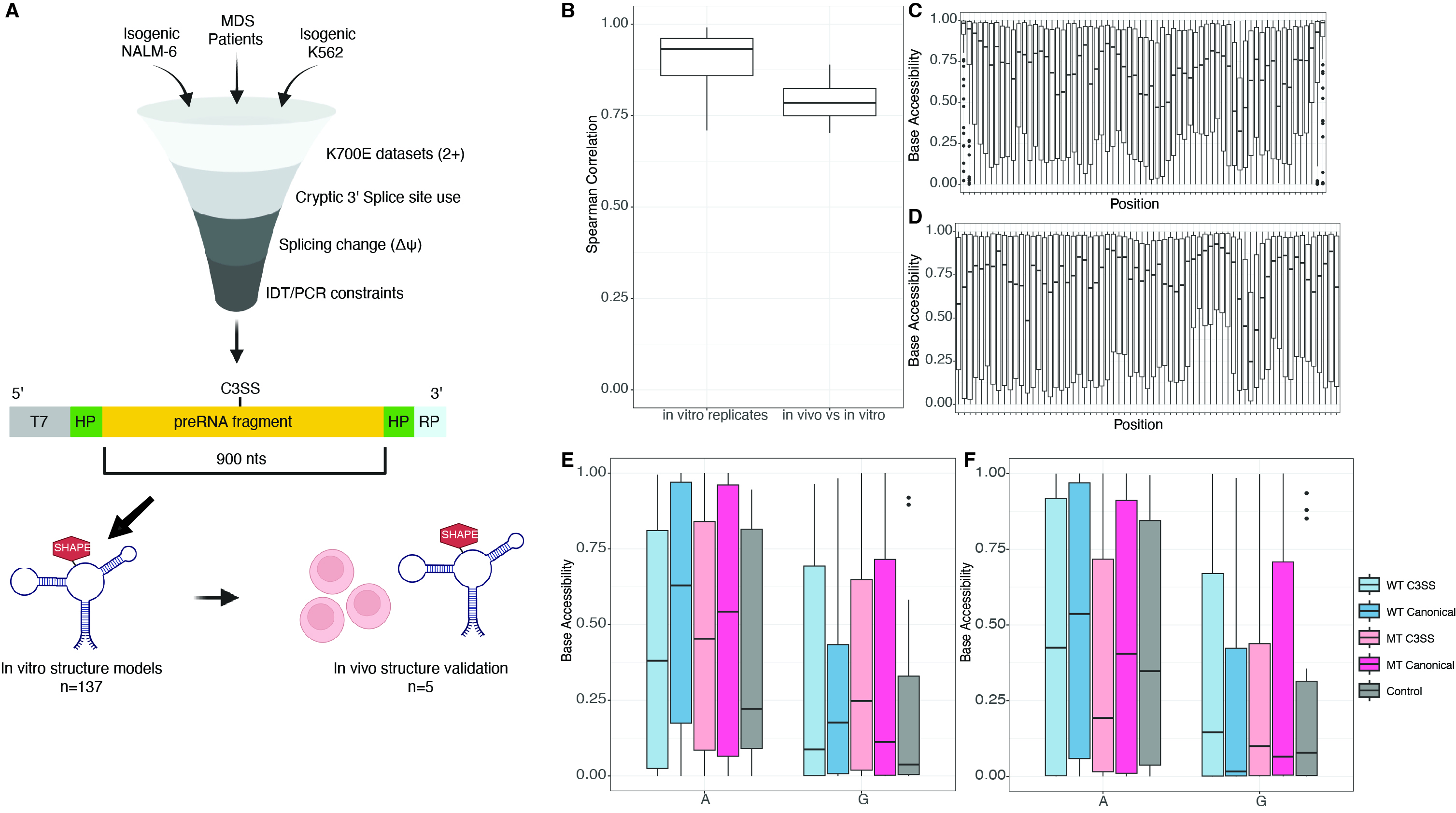

Supplement: Supplemental Figure 3 [file NIHMS2117327-supplement-Supplemental_Figure_3.jpg]

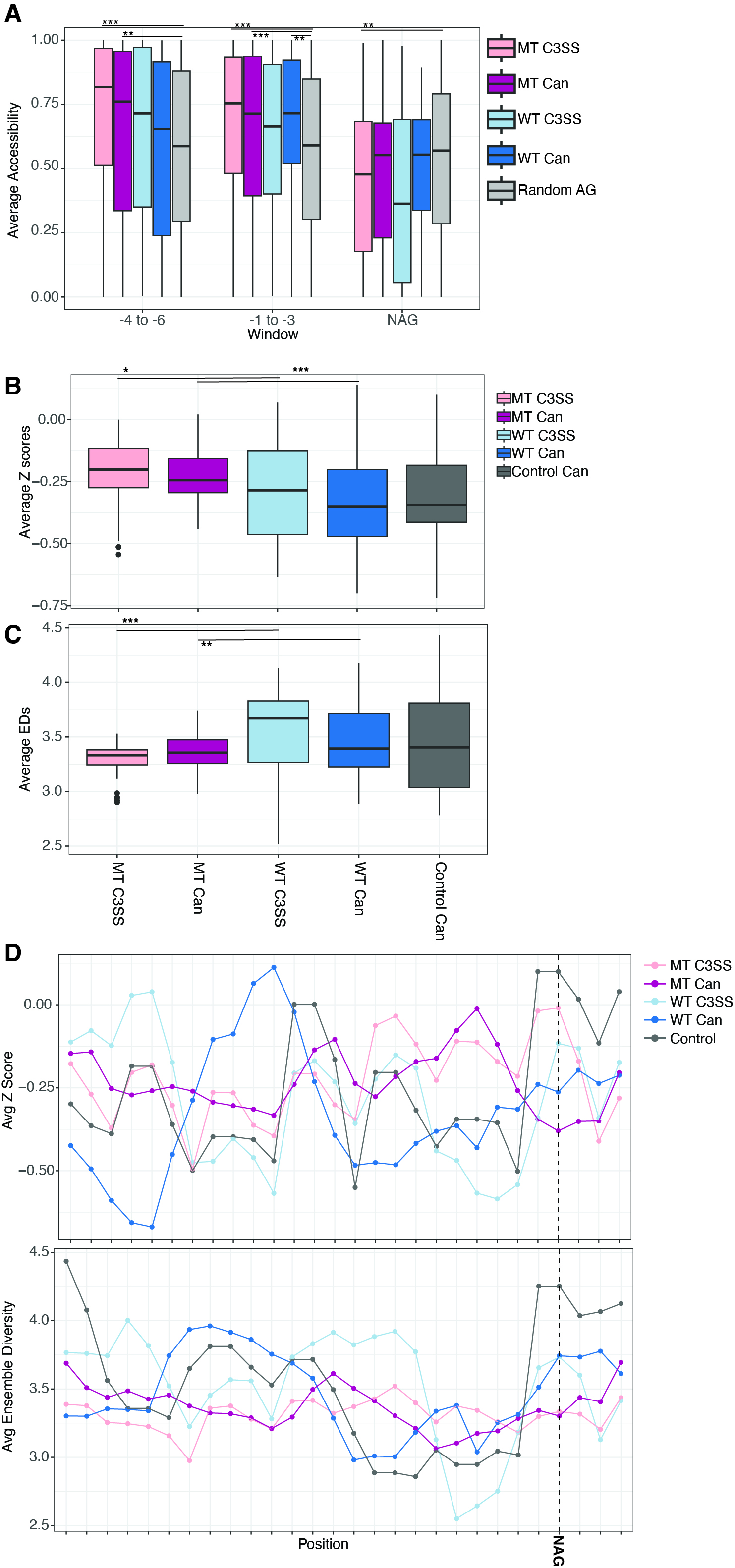

Supplement: Supplemental Figure 4 [file NIHMS2117327-supplement-Supplemental_Figure_4.jpg]

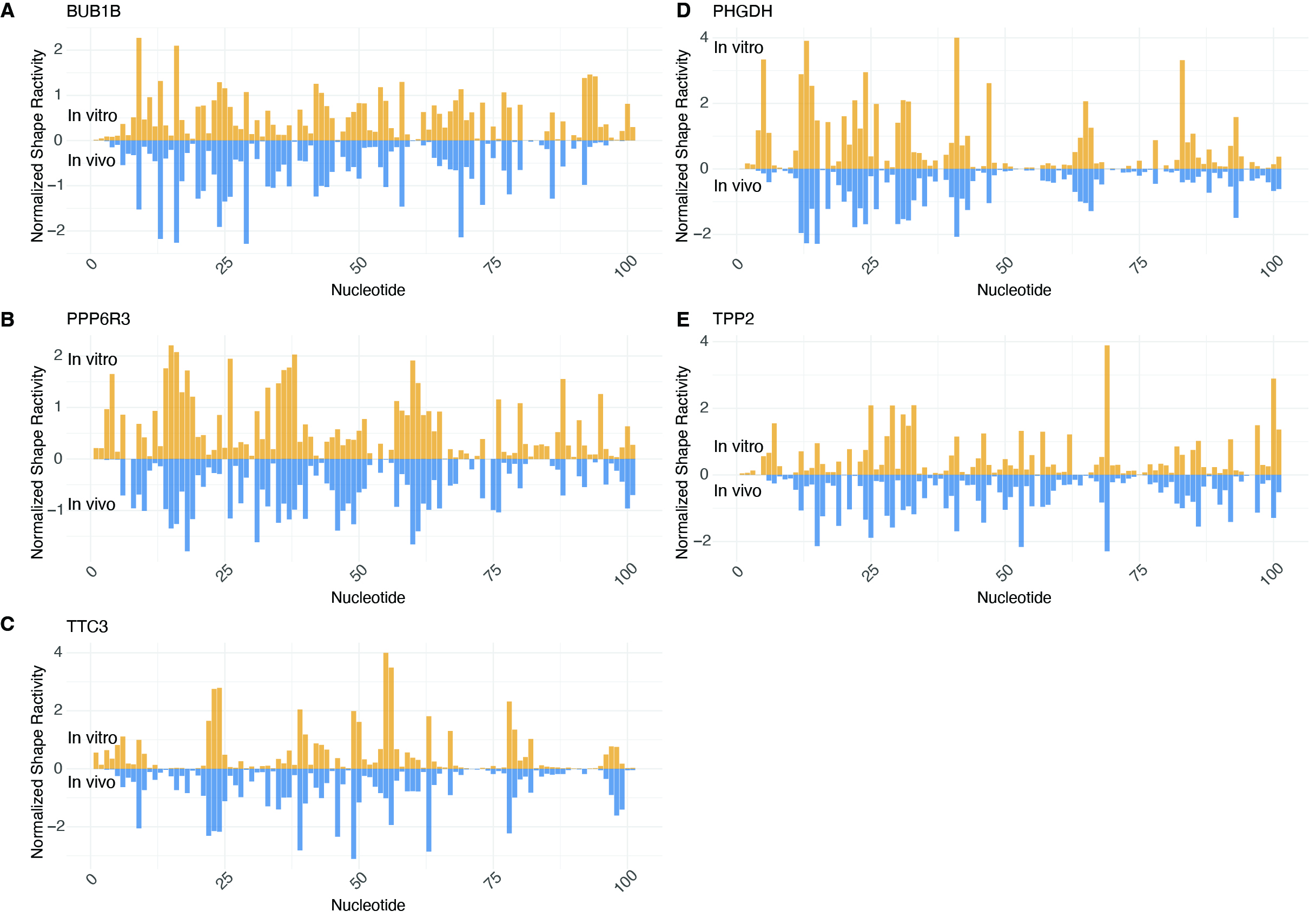

Supplement: Supplemental Figure 5 [file NIHMS2117327-supplement-Supplemental_Figure_5.jpg]

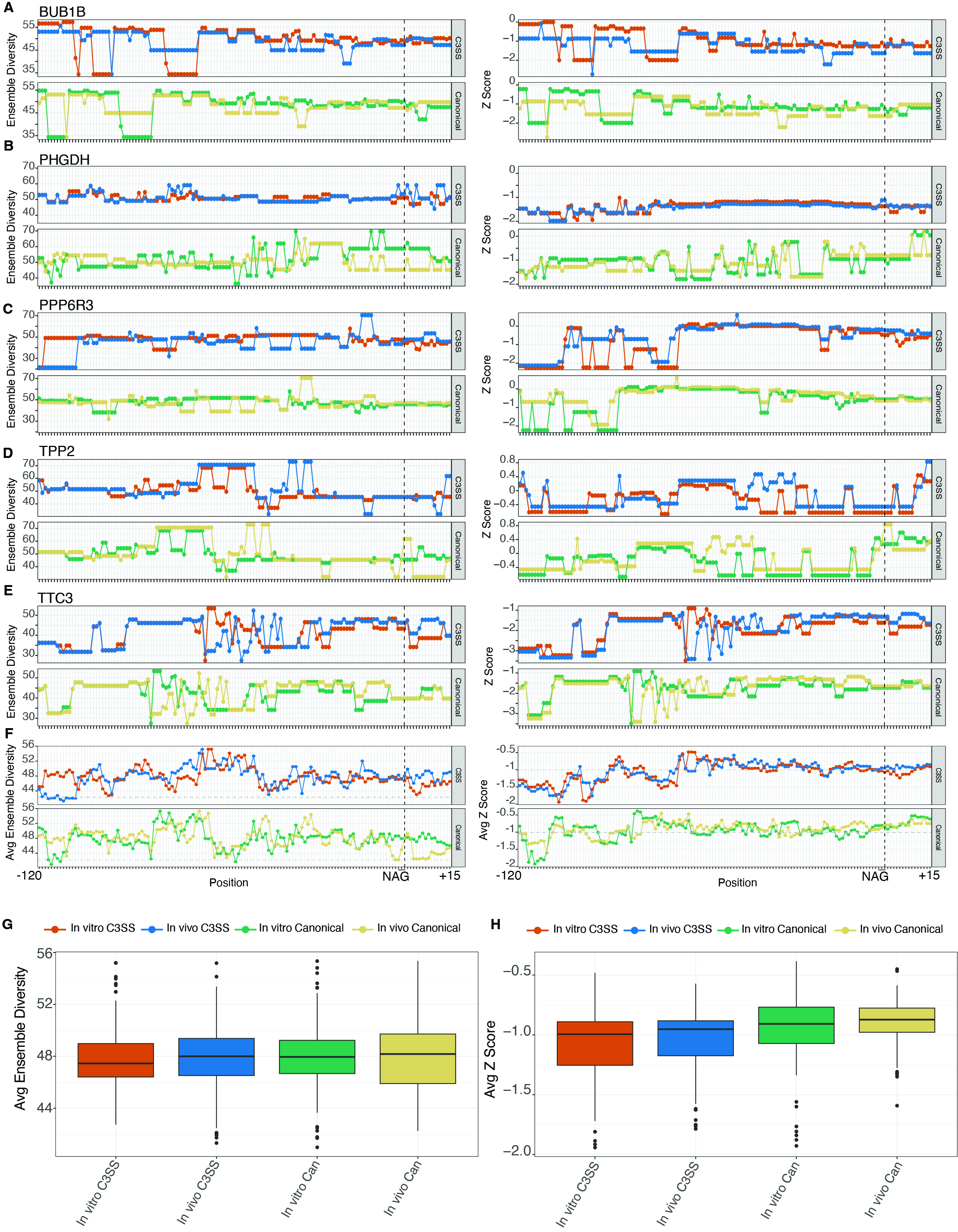

Supplement: Supplemental Figure 6 [file NIHMS2117327-supplement-Supplemental_Figure_6.jpg]
